# In-hospital survival characteristics and predictive model for patients with malignant tumors and sepsis

**DOI:** 10.3389/fmed.2026.1751311

**Published:** 2026-02-25

**Authors:** Ziyan Gan, Jiahao Zhang, Jinpeng Huang, Shunqin Long, Wanyin Wu, Guo Wang, Xiaobin Yao, Qiang Li, Xiaobin Yang, Yonglin Li

**Affiliations:** 1 Department of Oncology, The Second Affiliated Hospital of Guangzhou University of Chinese Medicine, Guangzhou, China; 2 Department of Information, The Second Affiliated Hospital of Guangzhou University of Chinese Medicine, Guangzhou, China; 3 Department of Emergency, The Second Affiliated Hospital of Guangzhou University of Chinese Medicine, Guangzhou, China; 4 Department of Emergency, The Second Clinical College of Guangzhou University of Chinese Medicine, The Second Affiliated Hospital of Guangzhou University of Chinese Medicine, Guangzhou, China

**Keywords:** machine learning, malignant tumors, prognostic factors, random forest, sepsis

## Abstract

**Objectives:**

To investigate the factors associated with in-hospital survival prognosis in participants with malignant tumors complicated by sepsis and to develop a predictive model.

**Methods:**

A retrospective study was conducted to collect data from 2,152 participants with malignant tumors complicated by sepsis, hospitalized at Guangdong Provincial Hospital of Chinese Medicine between January 2014 and June 2024. Univariate and multivariable logistic regression analyses were performed to identify independent risk factors, and the ADASYN oversampling technique was applied to address class imbalance. The dataset was randomly split into training and testing sets at an 8:2 ratio. Key features were selected using the recursive feature elimination (RFE) method, and eight machine learning models (logistic regression, decision tree, random forest, K-nearest neighbors, support vector machine, naive Bayes, stochastic gradient boosting, and neural network) were evaluated and hyperparameter-optimized.

**Results:**

A total of 2,152 participants were included in the study, with an in-hospital mortality rate of 12.6%. Multivariable analysis indicated that age, SOFA score, coagulation dysfunction, and metabolic abnormalities were important prognostic risk factors. The random forest model showed excellent discriminative ability on the validation set, with an AUC of 0.95, sensitivity of 91%, and specificity of 85%. A total of 10 features with the highest predictive value were selected using the RFE method, including troponin T, platelet distribution width, neutrophil count, red blood cell distribution width, fibrinogen, prothrombin time activity, aspartate transaminase, urea, low-density lipoprotein cholesterol, and creatinine.

**Conclusion:**

Age, SOFA score, coagulation dysfunction, and metabolic abnormalities are important prognostic risk factors for participants with malignant tumors complicated by sepsis. The random forest model constructed based on these key features has good predictive performance and can provide a powerful tool for the prognosis assessment of participants with malignant tumors complicated by sepsis. Future research needs to further validate the applicability and practical value of the model in different populations.

## Introduction

Sepsis is a life-threatening organ dysfunction caused by a dysregulated host response to infection (1). It has a high incidence and mortality rate, accounting for nearly 20% of global deaths, making it the leading cause of death from infection (2). Sepsis not only seriously threatens human health but also imposes a significant economic burden on society (3, 4). Early diagnosis and targeted treatment can significantly improve the prognosis of sepsis participants (5, 6).

In recent years, advancements in cancer treatment technologies have extended the survival time of malignant tumor participants (7). However, the immunosuppression caused by the disease and its treatment results in a significantly higher risk of infection in these participants compared to the general population (8). The coexistence of malignant tumors and sepsis not only increases the complexity of treatment but also significantly raises the risk of participant mortality (9–11).

Although several machine learning models have been developed to predict outcomes in general sepsis populations (12–14), none are specifically tailored to patients with malignant tumors, a group that exhibits distinct pathophysiological features, including therapy-induced immunosuppression, atypical infection presentation, and tumor-related metabolic and coagulopathic disturbances. Conventional sepsis severity scores such as SOFA were derived primarily from non-cancer intensive care cohorts and may not fully capture the unique risk profile of this vulnerable population. Therefore, a prognostic model that integrates cancer-specific biomarkers reflecting the interplay between inflammation, coagulation, and metabolic dysregulation is urgently needed to enable early identification of high-risk patients and support personalized clinical decision-making.

This study retrospectively analyzed the clinical data of 2,152 participants with both malignant tumors and sepsis. This study aimed to characterize survival-associated factors and develop a machine learning–based predictive model for in-hospital mortality among participants with malignant tumors complicated by sepsis.

## Materials and methods

### Study design and setting

This study retrospectively collected data from participants with malignant tumors and sepsis who were hospitalized at the Guangdong Provincial Hospital of Chinese Medicine from January 2014 to June 2024. The research protocol was approved by the Ethics Committee of the Guangdong Provincial Hospital of Chinese Medicine (approval number: BE2024-196-01). Given the retrospective nature of the study and the complete anonymization of participant data, the requirement for informed consent was waived.

### Study population and inclusion criteria

We included adult participants (aged ≥ 18 years) hospitalized with a confirmed diagnosis of both malignant tumor and sepsis during the study period. Sepsis was defined according to the Sepsis-3 criteria: a suspected or confirmed infection accompanied by an acute increase in the Sequential Organ Failure Assessment (SOFA) score of ≥2 points from baseline. In this study, “suspected infection” was operationally defined as the concurrent presence of: (1) blood culture collection, and (2) initiation of antimicrobial therapy for at least 48 consecutive hours, as documented in the electronic medical records. This definition was applied uniformly to all participants to ensure diagnostic consistency, particularly in the context of underlying malignancy where non-infectious systemic inflammation may mimic sepsis. Participants were excluded if they had incomplete baseline data or were lost to follow-up before discharge or death. All participant identifiers were removed prior to analysis to protect privacy.

### Data collection and variables

Demographic characteristics, comorbidities, and laboratory parameters were extracted from electronic health records. A total of 41 variables were collected, including: Demographics: age, gender; Comorbidities: hypertension, coronary heart disease, diabetes, chronic obstructive pulmonary disease, chronic kidney disease, cerebrovascular disease, chronic liver disease; Severity score: SOFA score; Laboratory markers: high-sensitivity C-reactive protein (hs-CRP), activated partial thromboplastin time (APTT), thrombin time (TT), international normalized ratio (INR), prothrombin time activity (PTA%), prothrombin time (PT), fibrinogen (FIB), white blood cell count (WBC), neutrophil count (NEUT), lymphocyte count (LYM), hemoglobin (Hb), hematocrit (HCT), red cell distribution width (RDW), platelet count (PLT), platelet distribution width (PDW), albumin (ALB), troponin T (TnT), low-density lipoprotein cholesterol (LDL-C), non-high-density lipoprotein cholesterol (non-HDL-C), high-density lipoprotein cholesterol (HDL-C), alanine aminotransferase (ALT), aspartate aminotransferase (AST), creatinine (Cr), potassium, sodium, urea, prealbumin (PA), total bilirubin (TBIL), direct bilirubin (DBIL), total cholesterol (TC), and total carbon dioxide (TCO_2_). All participants were followed until the primary endpoint: either in-hospital death or discharge. All laboratory measurements are reported in standard SI units (e.g., creatinine in μmol/L, fibrinogen in g/L, troponin T in ng/L, electrolytes in mmol/L).

### Statistical analysis and machine learning pipeline

All statistical analyses and machine learning procedures were implemented in Python (version 3.11). The analytical workflow consisted of the following steps:

Missing data handling: The proportion of missing values across all 41 predictor variables ranged from 5.5% to 22.1%. No variable exceeded a pre-specified exclusion threshold of 30% missingness; therefore, all variables were retained for imputation. Missing values in the predictors were addressed using Multiple Imputation by Chained Equations (MICE) with 20 imputed datasets under the missing-at-random (MAR) assumption. The outcome variable (in-hospital mortality) was complete and not imputed. Results were pooled using Rubin’s rules.

Class imbalance correction: After multiple imputation and dataset splitting, the Adaptive Synthetic Sampling (ADASYN) technique was applied only to the training set to address class imbalance in the minority class (non-survivors). ADASYN algorithm was used to address class imbalance. Unlike simple oversampling, ADASYN generates synthetic samples for the minority class with a focus on regions that are harder to learn, thereby improving classifier performance on critical cases.

Data partitioning: The original dataset was randomly split into training (80%) and test (20%) sets. All preprocessing steps, including imputation, ADASYN, and feature selection, were performed exclusively on the training set to prevent data leakage.

Feature selection: Recursive Feature Elimination (RFE) with cross-validation was applied to identify the most predictive subset of features from the 41 candidate variables.

Model development: Eight machine learning algorithms were trained and optimized: Logistic Regression (LR), Decision Tree (DT), Random Forest (RF), K-Nearest Neighbors (KNN), Support Vector Machine (SVM), Naive Bayes (NB), Stochastic Gradient Boosting (SGBT), and Neural Network (NNET). Hyperparameter tuning was performed via three rounds of 5-fold cross-validation using grid search, with area under the ROC curve (AUC) as the optimization metric. The specific hyperparameter grids searched for each algorithm are detailed in Supplementary Table 1.

Model evaluation: Final performance was assessed on the held-out test set using accuracy, AUC (with 95% confidence interval), sensitivity, specificity, calibration plots, precision-recall curves, confusion matrices, and prediction probability distributions. A schematic overview of the entire analytical pipeline is provided in Supplementary Figure 1.

Statistical testing: Descriptive statistics were presented as mean ± SD or median (IQR) for continuous variables, and frequencies (%) for categorical variables. Group comparisons used *t*-tests, Wilcoxon rank-sum tests, χ^2^ tests, or Fisher’s exact tests as appropriate. Univariate and multivariable logistic regression models were used to identify independent risk factors for in-hospital mortality. Results are reported as odds ratios (OR) with 95% confidence intervals (CIs). All analyses were two-tailed, with statistical significance set at α = 0.05.

## Results

### Demographic characteristics

The study included 2,152 participants (1,356 male, 796 female), with a mean age of 67.09 years (median: 68 years). The primary tumor types were gastrointestinal (*n* = 980), respiratory (*n* = 461), breast (*n* = 186), nasopharyngeal/oropharyngeal (*n* = 147), prostate (*n* = 108), urinary tract (*n* = 89), and skin malignancies (*n* = 12). Overall, 272 participants (12.6%) died during hospitalization. Compared to survivors, non-survivors were significantly older, had higher SOFA scores, and exhibited a greater burden of comorbidities, particularly cerebrovascular disease and diabetes. Laboratory profiles revealed more severe coagulopathy (elevated INR, PT, TT; reduced fibrinogen and PTA%), systemic inflammation (higher WBC and neutrophil count), metabolic derangement (elevated urea, reduced albumin, and HDL-C), and cardiac injury (elevated AST and troponin T), collectively indicating a more critical clinical state at presentation (Table 1).

**TABLE 1:** Demographic and clinical characteristics of the cohort.

Characteristics	Level	Overall	In-hospital survival	In-hospital death	*P*
*n*	Female	2,152	1,880	272	–
Gender (%)		796 (37.0)	689 (36.6)	107 (39.3)	0.429
Hypertension (%)	No	1,328 (61.7)	1,171 (62.3)	157 (57.7)	0.167
Coronary_artery_disease (%)	No	1,873 (87.0)	1,641 (87.3)	232 (85.3)	0.413
Diabetes_mellitus (%)	No	1,743 (81.0)	1,536 (81.7)	207 (76.1)	0.034
Chronic_obstructive_pulmonary_disease (%)	No	2,056 (95.5)	1,794 (95.4)	262 (96.3)	0.608
Chronic_kidney_disease (%)	No	2,025 (94.1)	1,773 (94.3)	252 (92.6)	0.343
Cerebrovascular_disease (%)	No	1,785 (82.9)	1,579 (84.0)	206 (75.7)	0.001
Liver_disease (%)	No	1,868 (86.8)	1,630 (86.7)	238 (87.5)	0.789
Tumor (%)	Malignant neoplasm of bone and articular cartilage	6 (0.3)	6 (0.3)	0 (0.0)	0.019
	Malignant neoplasm of breast	186 (8.7)	166 (8.9)	20 (7.4)	–
	Malignant neoplasm of digestive organs	980 (45.8)	872 (46.6)	108 (40.0)	–
	Malignant neoplasm of eye, brain, and other CNS	20 (0.9)	17 (0.9)	3 (1.1)	–
	Malignant neoplasm of female genital organs	120 (5.6)	110 (5.9)	10 (3.7)	–
	Malignant neoplasm of lip, oral cavity and pharynx	147 (6.9)	131 (7.0)	16 (5.9)	–
	Malignant neoplasm of male genital organs	108 (5.0)	88 (4.7)	20 (7.4)	–
	Malignant neoplasm of respiratory and intrathoracic organs	461 (21.5)	381 (20.4)	80 (29.6)	–
	Malignant neoplasm of thyroid and other endocrine glands	11 (0.5)	10 (0.5)	1 (0.4)	–
	Malignant neoplasm of urinary tract	89 (4.2)	80 (4.3)	9 (3.3)	–
	Melanoma and other malignant neoplasms of skin	12 (0.6)	9 (0.5)	3 (1.1)	–
SOFA (median [IQR])	–	4.00 [3.00, 6.00]	4.00 [3.00, 5.00]	5.00 [3.00, 9.00]	<0.001
Age (median [IQR])	–	68.00 [59.00, 77.00]	67.00 [59.00, 76.00]	71.00 [60.00, 80.00]	<0.001
hs-CRP (median [IQR])	–	80.77 [32.40, 145.38]	79.80 [31.86, 145.76]	83.61 [39.82, 142.82]	0.429
Activated_partial_thromboplastin_time (median [IQR])	–	36.00 [30.08, 41.90]	36.10 [30.20, 41.80]	35.70 [29.67, 42.30]	0.997
Thrombin_time (median [IQR])	–	17.20 [16.00, 18.60]	17.20 [16.00, 18.60]	17.50 [16.28, 19.40]	0.005
International_normalized_ratio (median [IQR])	–	1.16 [1.07, 1.30]	1.16 [1.06, 1.29]	1.21 [1.09, 1.43]	<0.001
Prothrombin_time_activity (median [IQR])	–	74.95 [61.58, 87.43]	75.50 [63.00, 88.00]	67.85 [52.98, 80.85]	<0.001
Prothrombin_time (median [IQR])	–	14.30 [13.00, 15.90]	14.20 [13.00, 15.80]	14.70 [13.17, 16.70]	0.003
Fibrinogen (median [IQR])	–	4.36 [3.14, 5.73]	4.47 [3.25, 5.81]	3.68 [2.48, 4.99]	<0.001
White_blood_cell_count (median [IQR])	–	9.15 [5.36, 14.12]	8.91 [5.15, 13.80]	11.39 [6.94, 17.24]	<0.001
Red_cell_distribution_width (median [IQR])	–	14.90 [13.50, 17.10]	14.70 [13.50, 16.90]	15.80 [14.07, 18.20]	<0.001
Lymphocyte_count (median [IQR])	–	0.80 [0.49, 1.21]	0.80 [0.50, 1.22]	0.78 [0.49, 1.17]	0.787
Hematocrit (median [IQR])	–	29.40 [24.17, 34.60]	29.60 [24.30, 34.62]	27.65 [22.87, 33.42]	0.013
Hemoglobin (median [IQR])	–	96.00 [78.00, 114.00]	97.00 [79.00, 115.00]	89.50 [74.75, 110.50]	0.006
Platelet_count (median [IQR])	–	177.00 [97.00, 274.25]	179.00 [99.00, 274.25]	161.50 [89.75, 274.50]	0.264
Platelet_distribution_width (median [IQR])	–	15.80 [13.07, 16.30]	15.80 [13.40, 16.30]	15.70 [12.10, 16.30]	0.193
Neutrophil_count (median [IQR])	–	7.44 [3.80, 12.31]	7.13 [3.64, 11.85]	9.52 [5.66, 15.29]	<0.001
Albumin (median [IQR])	–	31.65 [27.78, 35.80]	31.90 [27.90, 36.00]	30.15 [26.40, 34.50]	<0.001
Troponin_t (median [IQR])	–	0.02 [0.01, 0.05]	0.02 [0.01, 0.05]	0.04 [0.02, 0.09]	<0.001
Low_density_lipoprotein_cholesterol (median [IQR])	–	2.17 [1.51, 2.91]	2.19 [1.53, 2.94]	2.04 [1.36, 2.71]	0.005
Non-_high_density_lipoprotein_cholesterol (median [IQR])	–	2.78 [2.10, 3.57]	2.79 [2.12, 3.57]	2.64 [1.95, 3.60]	0.164
High_density_lipoprotein_cholesterol (median [IQR])	–	0.78 [0.52, 1.05]	0.79 [0.54, 1.06]	0.68 [0.42, 0.94]	<0.001
Alanine_aminotransferase (median [IQR])	–	23.00 [13.00, 45.00]	23.00 [13.23, 44.00]	26.00 [13.00, 64.32]	0.043
Aspartate_aminotransferase (median [IQR])	–	33.00 [21.00, 70.00]	32.00 [21.00, 66.00]	45.50 [24.00, 127.00]	<0.001
Creatinine (median [IQR])	–	80.00 [59.38, 121.00]	79.30 [60.00, 120.00]	84.50 [57.80, 137.50]	0.72
Potassium (median [IQR])	–	3.95 [3.56, 4.34]	3.94 [3.55, 4.33]	4.03 [3.60, 4.52]	0.019
Sodium (median [IQR])	–	136.00 [132.00, 140.00]	136.00 [132.00, 140.00]	136.98 [131.60, 141.00]	0.527
Blood_urea_nitrogen (median [IQR])	–	6.55 [4.49, 10.71]	6.31 [4.40, 10.21]	7.98 [5.14, 14.78]	<0.001
Prealbumin (median [IQR])	–	87.00 [46.00, 147.00]	89.00 [49.00, 150.25]	72.00 [35.00, 121.00]	<0.001
Direct_bilirubin (median [IQR])	–	7.40 [4.20, 19.42]	7.10 [4.10, 17.70]	9.35 [4.95, 27.33]	<0.001
Total_cholesterol (median [IQR])	–	3.64 [2.83, 4.50]	3.64 [2.86, 4.51]	3.58 [2.62, 4.38]	0.025
Total_bilirubin (median [IQR])	–	14.60 [9.10, 30.52]	14.50 [9.00, 29.42]	15.60 [10.30, 42.33]	0.016
Total_carbon_dioxide (median [IQR])	–	23.60 [20.40, 26.30]	23.60 [20.60, 26.40]	22.80 [18.95, 25.90]	0.005

### Independent risk factors for prognosis in cancer participants with sepsis

**FIGURE 1:**
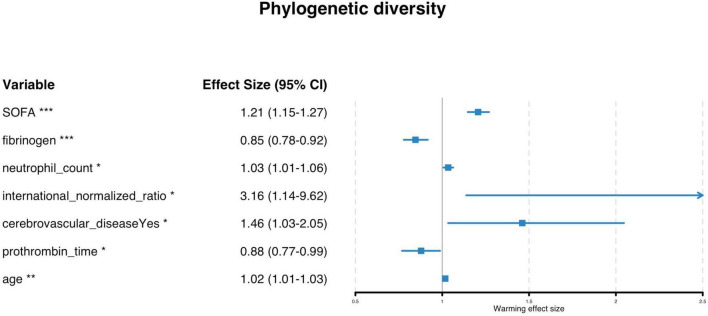
Adjusted odds ratios of independent risk factors for in-hospital mortality in cancer patients with sepsis (forest plot). The SOFA score demonstrated the strongest predictive value (highest significance: ***). Effect sizes marked with asterisks indicate statistical significance levels: ***P < 0.001, **P < 0.01, *P < 0.05. A cautionary note (“Warning effect size”) highlights potential uncertainty in interpreting the wide confidence interval for INR (1.14–9.62), suggesting limited precision for this estimate. The figure underscores the multifactorial nature of sepsis prognosis in cancer patients, integrating coagulation dysfunction, inflammatory markers, and comorbidities.

Univariate logistic regression identified SOFA score (OR = 1.24, 95% CI: 1.19–1.29), prothrombin time activity (OR = 0.98, 95% CI: 0.97–0.99), fibrinogen (OR = 0.80, 95% CI: 0.74–0.86), neutrophil count (OR = 1.04, 95% CI: 1.03–1.05), and white blood cell count (OR = 1.03, 95% CI: 1.02–1.04) as significant predictors (Supplementary Table 2). Multivariable analysis confirmed SOFA score (adjusted OR = 1.21, *P* < 0.001), fibrinogen, neutrophil count, INR, prothromin time, cerebrovascular disease, and age as independent risk factors. The strong effect of SOFA score highlights the pivotal role of organ dysfunction in mortality risk (Figure 1). Note that some ORs in Supplementary Table 2 are displayed as 1.00 due to rounding; unrounded values ranged from 0.995 to 1.004.

### Feature selection using ADASYN and RFE methods

**FIGURE 2:**
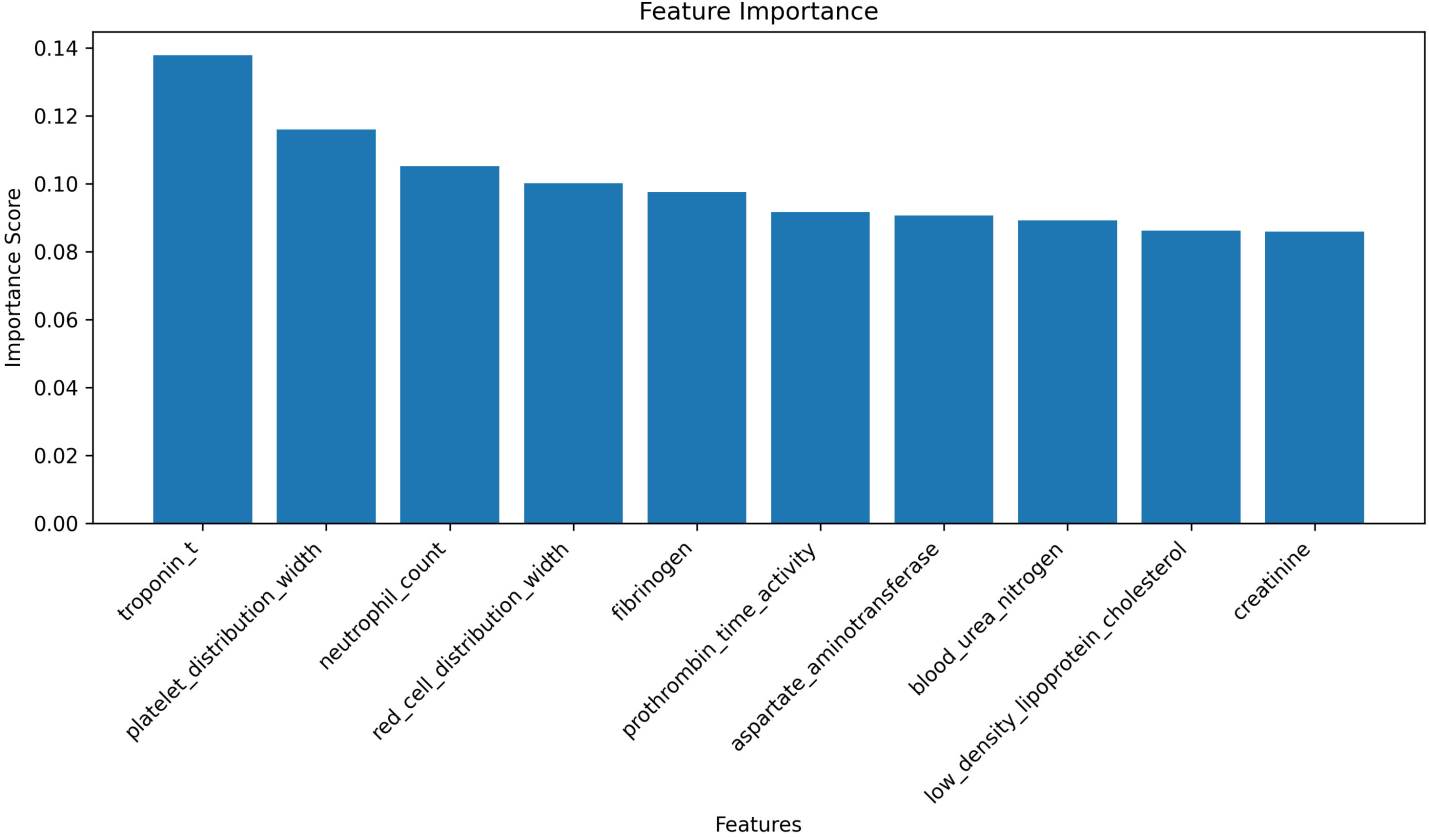
Feature importance ranking based on Recursive Feature Elimination (RFE). This bar chart illustrates the importance scores of 10 key predictive features selected through the Recursive Feature Elimination (RFE) method for predicting in-hospital mortality in cancer patients with sepsis. The importance scores range from 0.00 to 0.14.

After applying ADASYN to the training set to address class imbalance, the resampled dataset contained 3,818 cases (1,880 survivors and 1,938 non-survivors). Recursive Feature Elimination (RFE) identified the top 10 predictors: troponin T, platelet distribution width (PDW), neutrophil count (NEUT), red cell distribution width (RDW), fibrinogen (FIB), prothrombin time activity (PTA%), aspartate aminotransferase (AST), urea, low-density lipoprotein cholesterol (LDL-C), and high-sensitivity C-reactive protein (hs-CRP) (Figure 2). To assess feature stability, we performed three independent rounds of 5-fold cross-validation, yielding a mean AUC of 0.9389 (SD = 0.012).

### Model development and internal validation

Eight machine learning models were evaluated on the held-out test set. As shown in Table 2, Random Forest (RF) achieved the highest performance with an AUC of 0.88 (95% CI: 0.84–0.92), outperforming SGBT (AUC = 0.83), KNN (AUC = 0.82), and other algorithms. Naïve Bayes showed the lowest AUC (0.65).

**TABLE 2:** Model validation accuracy.

Model	Mean CV accuracy	Std CV accuracy	95% CI formatted
RF	0.88	0.02	0.88 (0.84, 0.92)
SGBT	0.83	0.02	0.83 (0.79, 0.87)
KNN	0.82	0.02	0.82 (0.78, 0.86)
SVM	0.80	0.02	0.80 (0.76, 0.84)
DT	0.78	0.02	0.78 (0.74, 0.82)
NNET	0.73	0.03	0.73 (0.67, 0.79)
LR	0.66	0.04	0.66 (0.58, 0.74)
NB	0.65	0.02	0.65 (0.61, 0.69)

### Performance of the optimal model

**FIGURE 3:**
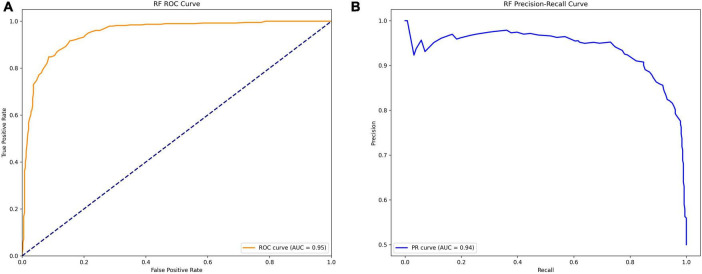
**(A)** Receiver Operating Characteristic (ROC) Curve and Area Under the Curve (AUC) of the random forest model. This figure presents the Receiver Operating Characteristic (ROC) curve of the Random Forest (RF) model on the validation set, evaluating its predictive performance for in-hospital mortality in cancer patients with sepsis. The x-axis represents the False Positive Rate (FPR), and the y-axis represents the True Positive Rate (TPR). The Area Under the Curve (AUC) is 0.95, indicating exceptional discriminative power. **(B)** Precision-Recall (PR) Curve and Area Under the Curve (AUC-PR) of the Random Forest Model. This figure illustrates the Precision-Recall (PR) curve of the Random Forest (RF) model on the validation set, evaluating its predictive performance for in-hospital mortality in cancer patients with sepsis. The x-axis represents Recall, and the y-axis represents Precision. The Area Under the Curve (AUC-PR) is 0.94, demonstrating strong discriminative power despite class imbalance (mortality rate: 12.6%).

**FIGURE 4:**
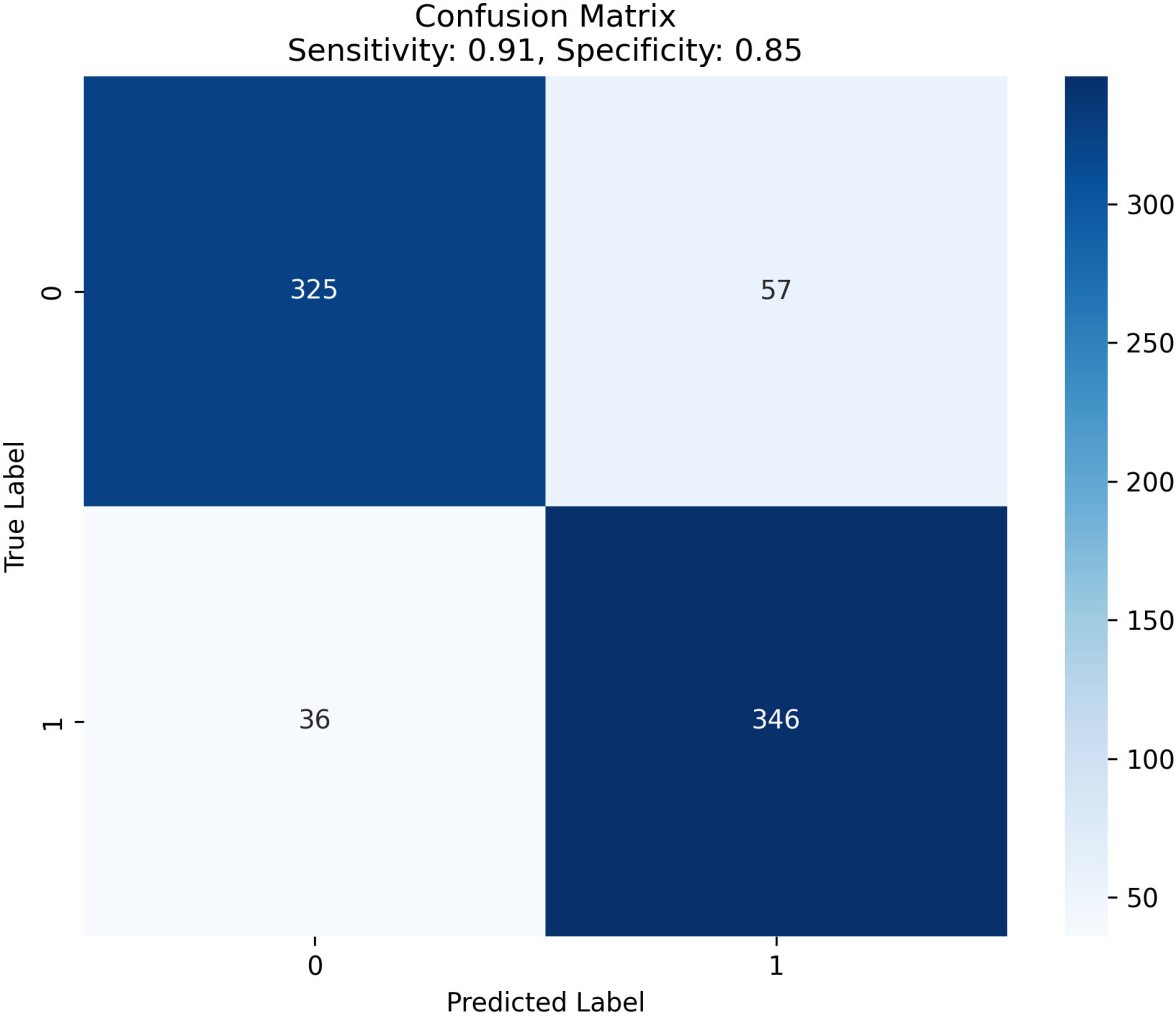
Confusion matrix with model sensitivity and specificity. This figure presents the confusion matrix performance metrics of the Random Forest model on the validation set, highlighting the model’s sensitivity (0.91) and specificity (0.85). The confusion matrix evaluates the classification accuracy for in-hospital mortality in cancer patients with sepsis by comparing True Labels (actual outcomes) against Predicted Labels (model predictions).

**FIGURE 5:**
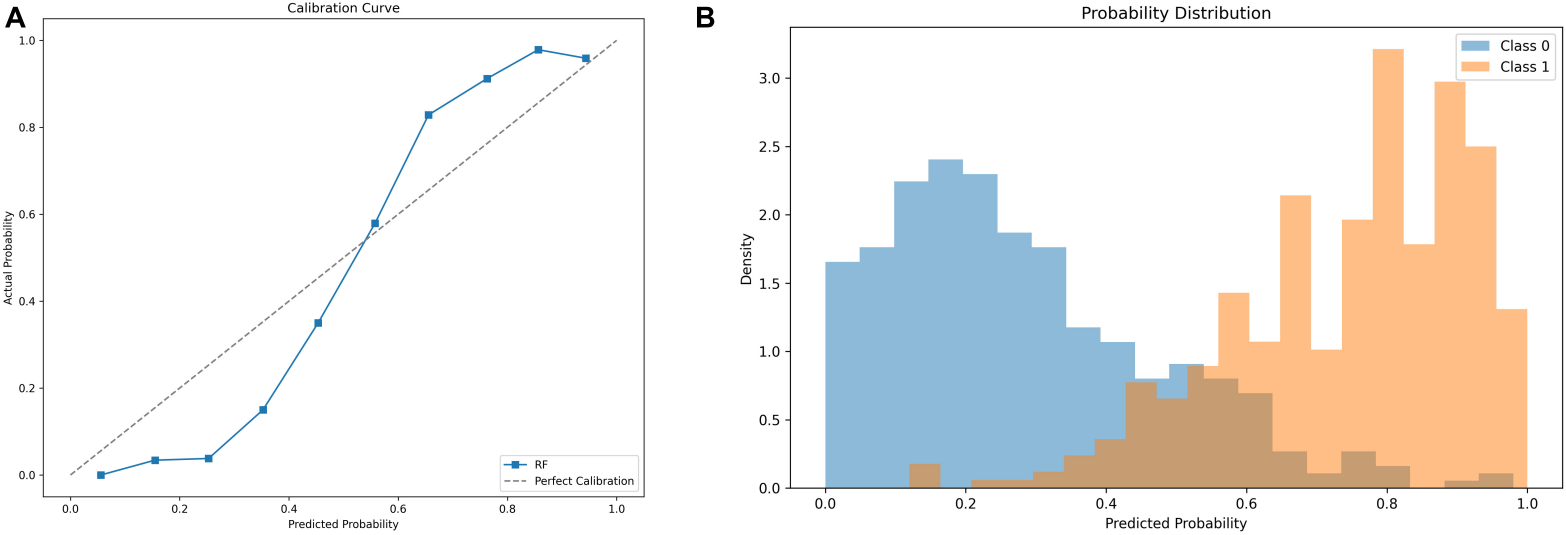
**(A)** Calibration curve of the Random Forest model vs. actual probability. This figure illustrates the calibration curve of the Random Forest (RF) model, evaluating the alignment between predicted probabilities and actual observed probabilities. **(B)** Probability distribution density plot of predicted outcomes. This figure illustrates the density distribution of predicted probabilities for in-hospital survival (Class 0) and in-hospital death (Class 1) in cancer patients with sepsis, as generated by the Random Forest model.

For benchmark comparison, a logistic regression model using the SOFA score as the sole predictor was evaluated on the same test set, achieving an AUC of 0.65 (95% CI: 0.61–0.68). The final RF model demonstrated excellent discrimination (AUC = 0.95, Figure 3A; PR-AUC = 0.94, Figure 3B), high sensitivity (91%) and specificity (85%) (Figure 4), and good calibration (Figure 5A). Predicted probabilities clearly separated survivors (80% < 0.4) from non-survivors (85% > 0.6) (Figure 5B), supporting its clinical utility for risk stratification.

## Discussion

This study analyzed clinical data from 2,152 cancer participants with sepsis to develop a predictive model for in-hospital mortality. Using logistic regression to identify independent prognostic factors combined with ADASYN and RFE methods, we selected 10 key predictors from 41 clinical indicators. Among eight machine learning models trained on these features, the optimal Random Forest model demonstrated excellent discriminative performance in the validation set (AUC = 0.95), with 91% sensitivity and 85% specificity. Three rounds of 5-fold cross-validation confirmed feature stability (mean accuracy 94.9%) and achieved effective mortality risk stratification despite extreme class imbalance (mortality rate 12.6%). This work provides an accurate prognostic tool for cancer participants with sepsis.

### Clinical characteristics of prognostic risk factors in cancer participants with sepsis

This study analyzed clinical data from 2,152 cancer participants with sepsis using logistic regression and identified several key prognostic risk factors: SOFA score, FIB, age, coagulation dysfunction, troponin T, neutrophil count, and comorbid diabetes. Compared to survivors, non-survivors were significantly older, had higher SOFA scores, and elevated neutrophil counts. These findings align with previous research by Zhu (15), which similarly identified advanced age, elevated SOFA scores, and increased inflammatory markers as significant prognostic indicators in this participant population.

Additionally, our study found that diabetes and cardiac injury were more prevalent among non-survivors, consistent with existing literature (16). Diabetes and cardiovascular disease may contribute to impaired immune function and vascular endothelial dysfunction, thereby increasing susceptibility to severe infections. Coagulation abnormalities also emerged as critical risk factors, with non-survivors exhibiting elevated INR, PT, and TT alongside reduced FIB and PT%, likely reflecting sepsis-induced disseminated intravascular coagulation (DIC) (17, 18). These results underscore the multifactorial nature of sepsis prognosis in cancer participants, highlighting the interplay between inflammation, metabolic dysregulation, and coagulopathy.

In terms of inflammation and metabolism, the in-hospital death group participants had significantly elevated levels of WBC, NEUT, Urea, and DBIL. Conversely, ALB, PA, and HDL-C were significantly reduced in this group. These changes indicate an enhanced inflammatory response and the presence of metabolic disorders, which further support the findings of previous studies (17, 19, 20).

### Integration of the coagulation–inflammation–metabolism axis in prognostic prediction

The 10 selected features collectively map onto a triad of pathophysiological axes central to sepsis outcomes in cancer patients: coagulation (fibrinogen, prothrombin time activity, platelet distribution width), inflammation (neutrophil count, red cell distribution width, hs-CRP), and metabolism (urea, LDL-C, AST). Fibrinogen and PTA% reflect sepsis-induced coagulopathy, which is often exacerbated by tumor procoagulant activity. PDW and RDW, which serve as markers of platelet and red blood cell heterogeneity, reflect underlying inflammatory stress and bone marrow dysregulation. Meanwhile, elevated urea and reduced LDL-C may indicate catabolic metabolism and impaired hepatic synthetic function, both common in advanced cancer. Notably, while SOFA score and troponin T are strong predictors of mortality, they largely serve as integrative markers of organ dysfunction severity rather than specific mechanistic drivers. Our model thus captures both the systemic burden of illness and cancer-specific biological perturbations, without implying causal relationships.

### Improvement in predictive performance of the multi-dimensional feature integration model

In this study, we adopted the Adaptive Synthetic Sampling (ADASYN) and Recursive Feature Elimination (RFE) methods. These methods were used to address the class imbalance problem and select key features. We ultimately selected 10 key predictors: Troponin T, neutrophil count, platelet distribution width, fibrinogen, red cell distribution width, urea nitrogen, creatinine, alanine aminotransferase, prothrombin time, and hypersensitive C-reactive protein. The random forest model demonstrated excellent discriminative ability on the validation set, with an AUC of 0.95, a sensitivity of 91%, and a specificity of 85%. Compared to traditional scoring systems, the multi-dimensional feature integration model more comprehensively captures the overall condition of participants. The ADASYN method effectively addressed the class imbalance problem by generating synthetic samples, thereby improving the model’s recognition ability for the minority class. The RFE method, by ranking the importance of features, retained the most predictive features, thus enhancing the overall performance of the model.

### Interaction of the coagulation-inflammation-metabolism axis in prognostic prediction

Our study delved into the interaction of the coagulation-inflammation-metabolism axis in prognostic prediction. We examined multiple indicators, including coagulation dysfunction (e.g., fibrinogen, prothrombin time activity), metabolic abnormalities (e.g., low-density lipoprotein cholesterol, urea), and organ damage (e.g., aspartate aminotransferase, direct bilirubin). These indicators collectively form a complex prognostic network.

Platelet parameters, such as platelet distribution width, reflect the functional state of platelets. Abnormal values in these parameters may be related to thrombosis or bleeding risk (21–24). Abnormal bilirubin metabolism, such as elevated direct bilirubin, indicates liver function impairment. Lipoprotein abnormalities, such as HDL and LDL, may be associated with inflammatory responses and decreased immune function (20, 25). The synergistic effect of these novel biomarkers further enhances the predictive power of the model.

### Potential clinical applications and implementation considerations

While our random forest model demonstrates strong predictive performance in this retrospective cohort, its clinical deployment remains hypothetical and requires rigorous prospective validation. The model could support risk-stratified care pathways, such as triggering early warning alerts for high-risk patients, guiding intensity of monitoring or intervention, or facilitating timely palliative care discussions in those with very poor predicted survival. However, we emphasize that these applications are not yet ready for real-world implementation. Several practical barriers must be addressed before bedside adoption, including seamless integration into electronic health record (EHR) systems, provision of interpretable outputs (e.g., through SHAP or LIME methods) to foster clinician trust, and demonstration of improved patient-centered outcomes, not just statistical performance in prospective interventional studies. Until such evidence is generated, the model should be viewed as a research tool rather than a clinical decision-making instrument.

### Limitations of the study

This study has several important limitations that must be acknowledged. First and foremost, this is a single-center retrospective study. The high discriminative performance (AUC = 0.95) reported here is derived from internal validation and is highly susceptible to overfitting and optimistic bias; as such, it must be interpreted with extreme caution. This model is a proof-of-concept and requires rigorous external validation in multi-center, prospective cohorts before any consideration for clinical application. Second, although the ADASYN oversampling technique was used to address class imbalance, the synthetic samples generated may not fully represent the true biological distribution of the minority class and could potentially introduce new biases. Third, our cohort encompasses a wide spectrum of malignancies, which have vastly different biologies, treatment regimens, and baseline prognoses. This heterogeneity, while increasing the generalizability of our model across cancer types in a broad sense, may also mask tumor subtype-specific risk patterns and affect the model’s performance in specific populations. Future studies should explore stratified analyses by cancer type or develop malignancy-specific models. Additionally, the study primarily focused on admission clinical characteristics and laboratory indicators, without considering the dynamic impact of subsequent treatment interventions (e.g., antibiotics, source control, intensive care support), which can significantly influence participant survival in real-world clinical settings. Future research should incorporate multicenter data, account for treatment variables, and explore different feature selection methods to further enhance the model’s accuracy, robustness, and practical utility.

## Conclusion

In this study, we analyzed clinical data from participants with malignant tumors and sepsis to construct a random forest prediction model based on multi-dimensional feature integration. The model demonstrated excellent discriminative ability in the validation set, offering a new tool for prognostic assessment in these participants. Future research should focus on validating the model’s applicability across different populations and medical environments. Additionally, the practical application value of the model in clinical practice should be further explored.

## Data Availability

The data analyzed in this study is subject to the following licenses/restrictions: requests for data access should be directed to the corresponding authors and will be considered on a case-by-case basis in accordance with institutional and legal requirements. Requests to access these datasets should be directed to Yonglin Li, liyonglin@gzucm.edu.cn.
