# Supplementary figures and images for: In-hospital survival characteristics and predictive model for patients with malignant tumors and sepsis
Source: Front Med (Lausanne). 2026 Feb 25;13:1751311. doi: 10.3389/fmed.2026.1751311 (PMC12975745; doi:10.3389/fmed.2026.1751311)

# Research Analysis Workflow

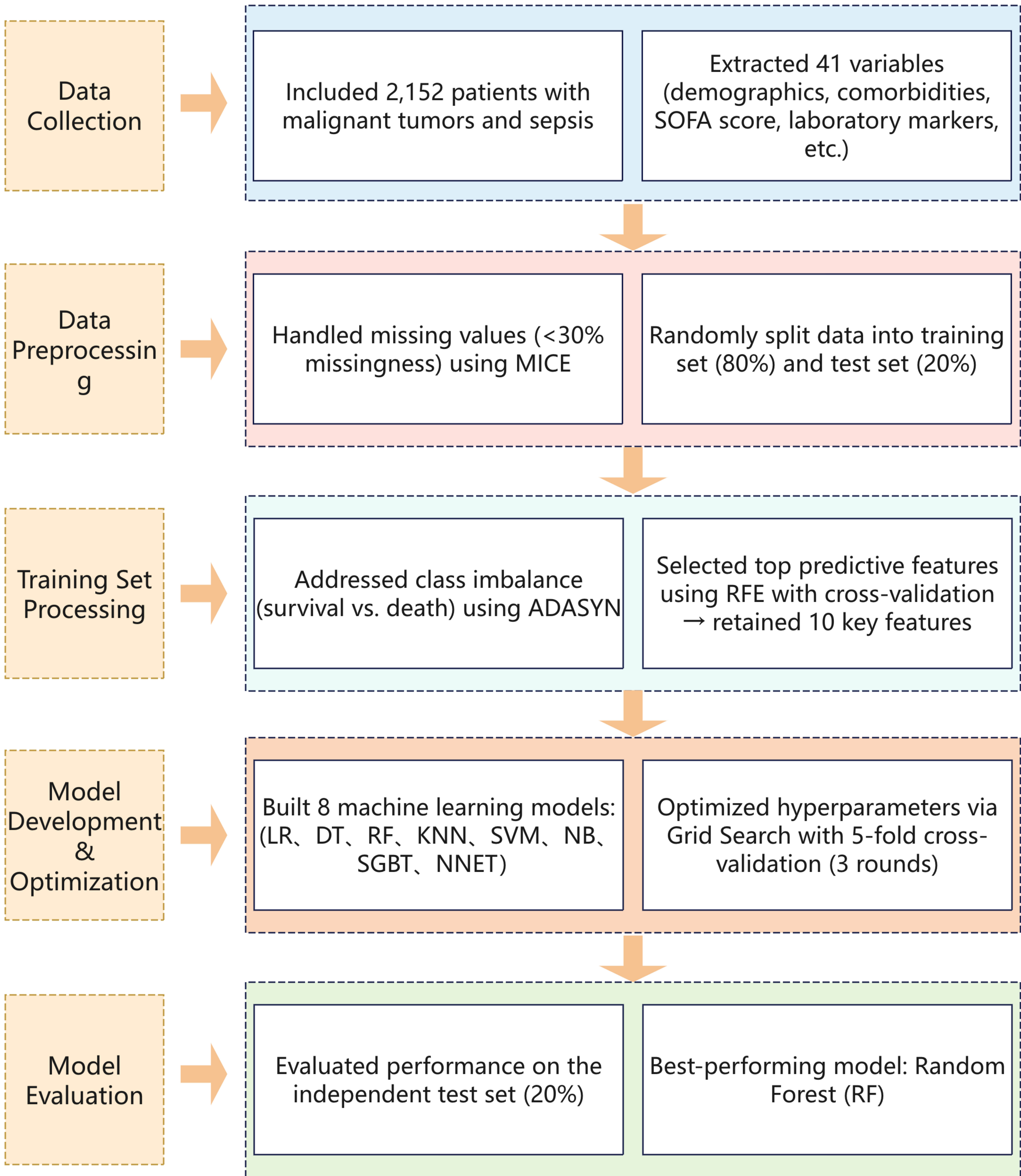

Supplement: Supplementary file 1 [file Data_Sheet_1.pdf]
